# Diseases of the Eye

**Published:** 1894-04-28

**Authors:** 


### DISEASES OF THE EYE.

*Electric Ophthalmia.*—Snell, in the *Medical Annual*,[1] gives an interesting account of this condition, which is produced by exposure of the eyes to the influence of the chemical rather than the heat rays which radiate from the electric arc as used in the process of "electrical welding." He describes the apparatus which is used to protect the worker as a sort of helmet covering the face and head, provided with a window, the glass of which consists of six layers of glass alternately blue and red in colour. The symptoms usually found in this form of ophthalmia are pain, photophobia, and inability to open the eyes. The condition is as a rule relieved by the application of cocaine.

*Conjunctiva.*—The discussion at the Ophthalmological Society on Mr. Jonathan Hutchinson's paper on "School Ophthalmia"[2] showed that there is a very considerable diversity of opinion as to the causation of granular ophthalmia. Most of the speakers agreed that there had been a marked increase in the number of cases of contagious and mild purulent ophthalmia, but the majority, whilst admitting that these cases if neglected might become trachomatous, looked upon the initial attack as not necessarily one of granular ophthalmia. Mr. Hutchinson laid down the following propositions as "memoranda for the guidance of school authorities"[3]: (*a*) A certificate of freedom from ophthalmia should always be required on the return of a pupil to school; (*b*) in the case of anyone in a school suffering from "sore eyes," isolation should be immediately adopted and advice obtained; (*c*) if in any case relapses occur and granular lids are developed, the isolation and treatment ought to be prolonged, and the pupil ought not to be allowed to return to school until the cure has been established (without relapse) for several months; (*d*) no case of granular lids, still requiring treatment, should ever be admitted into a school where there are healthy children; (*e*) in case of an outbreak in a school the treatment should be conducted on the spot, and the pupils ought not to be sent home; (*f*) the treatment need not interfere materially with school routine. The chief difficulty in carrying out these suggestions lies in the first, Who is to sign the certificate? With the remainder most will agree.

Burchardt[4] in writing on the treatment of acute gonorrhœal ophthalmia, condemns the use of cold compresses and applications of strong nitrate of silver to the mucous membrane. He advocates free irrigation of the conjunctival sacs with chlorine water of 5 per cent. strength, this is applied drop by drop between the lids, the head being held back, and the lids meanwhile manipulated so as to cause the solution to reach every part of the conjunctival folds. He follows this up by the application in the same way of a 1-10th per cent. solution of nitrate of silver.

Truc[5] contributes a paper on the contagiousness of trachoma. He says that "The risk of contagion is directly proportional to the amount and virulence of the conjunctival secretion." In France trachoma is to be regarded as a contagious disease only in the case of young lymphatic subjects, the affection being communicated by direct or indirect contact with a catarrhal or purulent conjunctival discharge. He sums up his conclusions briefly. "Granular ophthalmia is contagious, but less so than formerly. The affection is almost exclusively communicated from one member of a family to another, and by indirect contact with infective material. The moist types of the disease associated with a mucous or purulent secretion, are alone dangerous. Women, children, and persons of a lymphatic constitution are specially liable to infection. Isolation is always to be recommended, and should be insisted on in the case of the moist, and especially of the purulent variety of the affection."

In the treatment of blepharitis Valude[6] avoids the use of salves in the ulcerated form, and advises closure of the lids three or four times a day for half an hour with compresses saturated with strong solution of acetate of lead. Deep ulcers he touches up with mitigated silver nitrate, shallow ulcers he brushes over with 2 per cent. solution.

*Opthalmological Therapeutics.*—Schmitz[7] has recorded two cases of carbonic oxide poisoning with eye symptoms. In the first the patient was unconscious for twenty-four hours, and his limbs paralysed for three days. He suffered from intense photophobia, vision reduced to one-sixth, field of vision contracted. Symptoms gradually disappeared till at the end of five months, when all was again normal. In the second case there was also concentric contraction of the fields with lessened central visual acuteness. He regards both as cases of toxic hysteria.

A severe case of cocaine poisoning from a small dose of the drug is mentioned by A. R. Baker.[8] He was operating for lachrymal stricture, and after instilling two drops of 6 per cent. solution he slit the canaliculus, and injected three more drops into the sac. Finding the parts still very sensitive he injected three more, when almost immediately the patient became violently convulsed and unconscious for two hours. Then maniacal delirium set in and lasted for six to eight hours. At the end of that time she completely recovered.

Snell[9] contributes a valuable paper on the amblyopia produced in those engaged in the manufacture of explosives when di-nitrobenzol is used. He quotes five cases, in all of which most careful observations were made, and reproduces charts of the fields of vision. The symptoms shown were failure of sight, concentric contraction of the fields of vision with, in some cases, central scotoma. Enlargement of the retinal vessels, especially the veins, slight blurring of the edges of the disc and varying degrees of pallor. The amblyopia was accompanied by numbness of the extremities and unsteadiness of gait.

Scopolamine, according to Bellarininow,[10] is likely in the future to replace atropine in ophthalmic practice. It is said to act very quickly and completely as a mydriatic for the determination of refractive errors, and yet the period of paralysis of accommodation is very short. For this reason it is applicable as a substitute for atropine only in short-lived inflammatory attacks.

It has long been known that where corrosive sublimate is used in contact with a cornea anæsthetised by cocaine there is apt to follow considerable opacity. Mellinger[11] has experimentally shown that this opacity is due to the action of the corrosive sublimate on the parenchyma of the cornea, and that this action is rendered more complete by the cocaine facilitating its entrance. In a later research the same investigator has pointed out a further deleterious action of cocaine in the prevention of primary union of corneal wounds. He publishes the result of experiments on rabbits' eyes, corneal sections being made with and without cocaine anæsthesia. He finds that the cocaine prevents primary healing by hindering the formation of a coagulation support in the parenchyma, the healing being more or less epithelial and apt to separate. As a result of his researches, he urges that the instillation of cocaine should not be prolonged or needlessly repeated.

[1] Med. Annual, 1894. [2] Brit. Med. Journ., March 17, 1894. [3] Brit. Med. Journ., Feb. 10, 1894. [4] Brit. Med. Journ., Jan. 6, 1894. [5] The Med. Week, Dec. 22, 1874. [6] L'Union Medicale, No. 38, 1893. [7] Annales d'Oculistique, Nov., 1893. [8] American Journal of Ophthalmology, Nov., 1893. [9] Brit. Med. Journ., March 3, 1894. [10] Ed. Med. Journ., Jan., 1894. [11] Arch. fur Ophtholmologie, xxxvii., p. 159; Therap. Gazette, p. 827.

